# Crystal structures of *N*-(pyridin-2-ylmeth­yl)pyrazine-2-carboxamide (monoclinic polymorph) and *N*-(pyridin-4-ylmeth­yl)pyrazine-2-carboxamide^1^

**DOI:** 10.1107/S1600536814009519

**Published:** 2014-06-23

**Authors:** Dilovan S. Cati, Helen Stoeckli-Evans

**Affiliations:** a Debiopharm International S.A., Chemin Messidor 5-7, CP 5911, CH-1002 Lausanne, Switzerland; b Institute of Physics, University of Neuchâtel, rue Emile-Argand 11, CH-2000 Neuchâtel, Switzerland

**Keywords:** crystal structure, pyrazine, pyridine, carboxamide

## Abstract

The title compounds, C_11_H_10_N_4_O (H*L*1) and C_11_H_10_N_4_O (H*L*2), are pyridine 2-ylmethyl and 4-ylmethyl derivatives, respectively, of pyrazine-2-carboxamide. H*L*1 was measured at 153 K and crystallized in the monoclinic space group *P*2_1_/*c* with *Z* = 4. There has been a report of the same structure measured at room temperature but assumed to crystallize in the triclinic space group *P*-1 with *Z* = 4 [Sasan *et al.* (2008 ▸). *Monatsh. Chem.* **139**, 773–780]. In H*L*1, the pyridine ring is inclined to the pyrazine ring by 61.34 (6)°, while in H*L*2 this dihedral angle is 84.33 (12)°. In both mol­ecules, there is a short N—H⋯N inter­action involving the pyrazine carboxamide unit. In the crystal of H*L*1, mol­ecules are linked by N—H⋯N hydrogen bonds, forming inversion dimers with an *R*_2_^2^(10) ring motif. The dimers are linked *via* bifurcated-acceptor C—H⋯O hydrogen bonds, forming sheets lying parallel to (102). The sheets are linked *via* C—H⋯N hydrogen bonds, forming a three-dimensional structure. In the crystal of H*L*2, mol­ecules are linked by N—H⋯N and C—H⋯N hydrogen bonds to form chains propagating along [010]. The chains are linked *via* C—H⋯O hydrogen bonds, forming sheets lying parallel to (100). Within the sheets there are π–π inter­actions involving neighbouring pyrazine rings [inter-centroid distance = 3.711 (15) Å]. Adjacent sheets are linked *via* parallel slipped π–π inter­actions involving inversion-related pyridine rings [inter-centroid distance = 3.6395 (17) Å], forming a three-dimensional structure.

## Abstract 2

The title molecules, H*L*1 and H*L*2, differ in their conformation with the pyridine ring being inclined to the pyrazine ring by 61.34 (6) and 84.33 (12)°, respectively. The crystal packing is also slightly different, with molecules of H*L*1 linked by pairs of N—H⋯N hydrogen bonds, forming inversion dimers, while for H*L*2 molecules are linked by N—H⋯N and C—H⋯N hydrogen bonds, forming chains along [010].

## Chemical context   

The title compounds form part of a series of ligands synthesized in order to study their coordination chemistry with 3*d* transition metals (Cati, 2002 ▸).
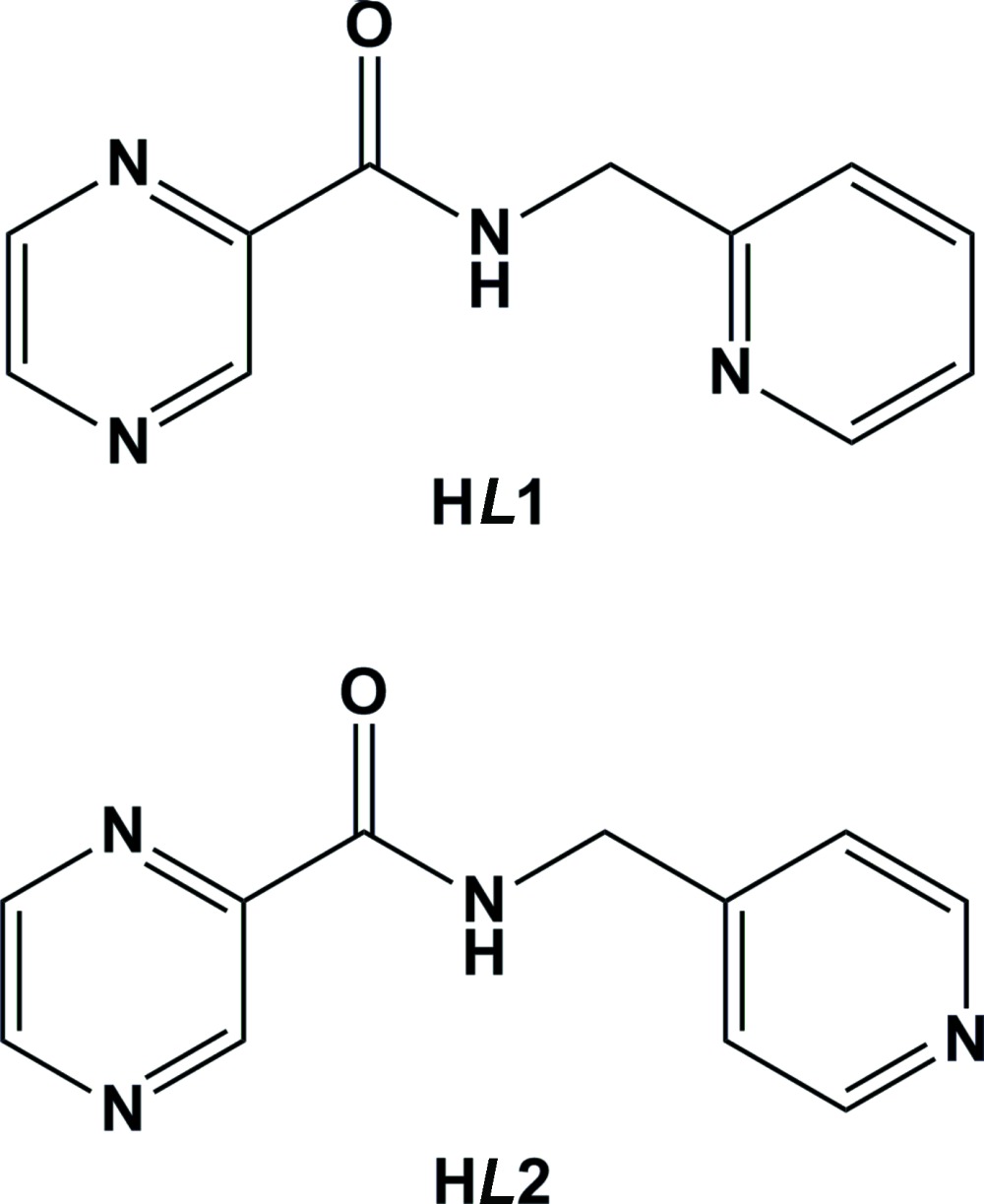


They have been used to construct coordination polymers and multi-nuclear compounds, and to study their magnetic properties (Cati *et al.*, 2004 ▸). Similar ligands have been synthesized by other groups who have studied, for example, the magnetic properties of some copper(II) complexes (Hausmann *et al.*, 2003 ▸; Kingele *et al.*, 2007 ▸). 

## Structural commentary   

**Figure 1:**
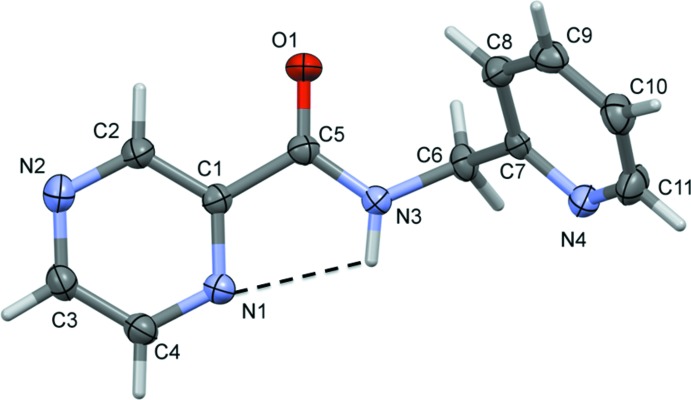
A view of the mol­ecular structure of H*L*1, with atom labelling. Displacement ellipsoids are drawn at the 50% probability level. The short intra­molecular N—H⋯N inter­action is shown as a dashed line (see Table 1 ▸ for details).

The mol­ecular structure of ligand H*L*1 is illustrated in Fig. 1 ▸. H*L*1 was measured at 153 K and crystallized in the monoclinic space group *P*2_1_/c with *Z* = 4. The β angle is 91.461 (11)° and the systematic absences, the *R_i_*_nt_ value (0.0348) and the successful refinement {*R*1 [*I* > 2σ(*I*)] = 0.0319} clearly show that at 153 K the space group is monoclinic *P*2_1_/c. The same structure measured at room temperature was reported to crystallize in the triclinic space group *P* 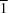 with *Z* = 4 (Sasan *et al.*, 2008 ▸). However, the three cell angles are close 90 (2)° [α = 91.802 (6), β = 89.834 (7), γ = 91.845 (6)°] and the crystal used was a very narrow needle. The final *R*1 [*I* > 2σ(*I*)] factor was rather high at 0.0699, hence it is possible that the choice of crystal system and space group are incorrect. However, this could not be confirmed when analysing the coordinates using the AddSymm routine in *PLATON* (Spek, 2009 ▸).

In the mol­ecule of H*L*1 there is a short N—H⋯N hydrogen bond present in the pyrazine carboxamide unit (Table 1 ▸), and the amide group, C5(=O1)N3, is approximately coplanar with the pyrazine (N1/N2/C1–C4) ring [dihedral angle = 2.56 (14)°]. The pyrazine and pyridine (N4/C7–C10) rings are inclined to one another by 61.34 (6)°. In the triclinic structure mentioned above, the same angle in the two independent mol­ecules is 63.31 (13) and 61.94 (13)°.

**Figure 2:**
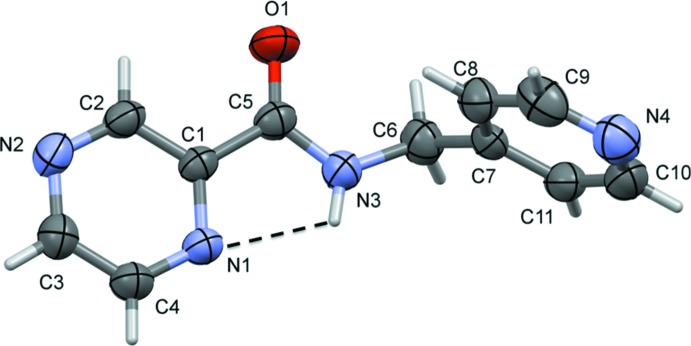
A view of the mol­ecular structure of H*L*2, with atom labelling. Displacement ellipsoids are drawn at the 50% probability level. The short intra­molecular N—H⋯N inter­action is shown as a dashed line (see Table 2 ▸ for details).

The mol­ecular structure of H*L*2 is illustrated in Fig. 2 ▸. Here too there is a short intra­molecular N—H⋯N contact involving the pyrazine carboxamide unit (Table 2 ▸), and the amide group, C5(=O1)N3, is almost coplanar with the pyrazine (N1/N2/C1–C4) ring with a dihedral angle of 3.9 (3)°. Here the pyrazine and pyridine (N4/C7–C10) rings are almost normal to one another with a dihedral angle of 84.33 (12)°.

## Supra­molecular features   

**Figure 3:**
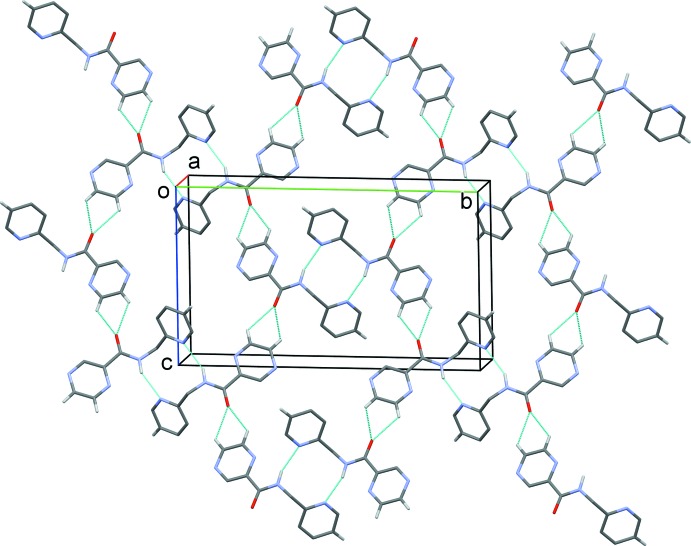
A partial view along the *a* axis of the crystal packing of compound H*L*1. The N—H⋯N, C—H⋯O and C-H⋯N hydrogen bonds are shown as dashed lines (see Table 1 ▸ for details).

**Figure 4:**
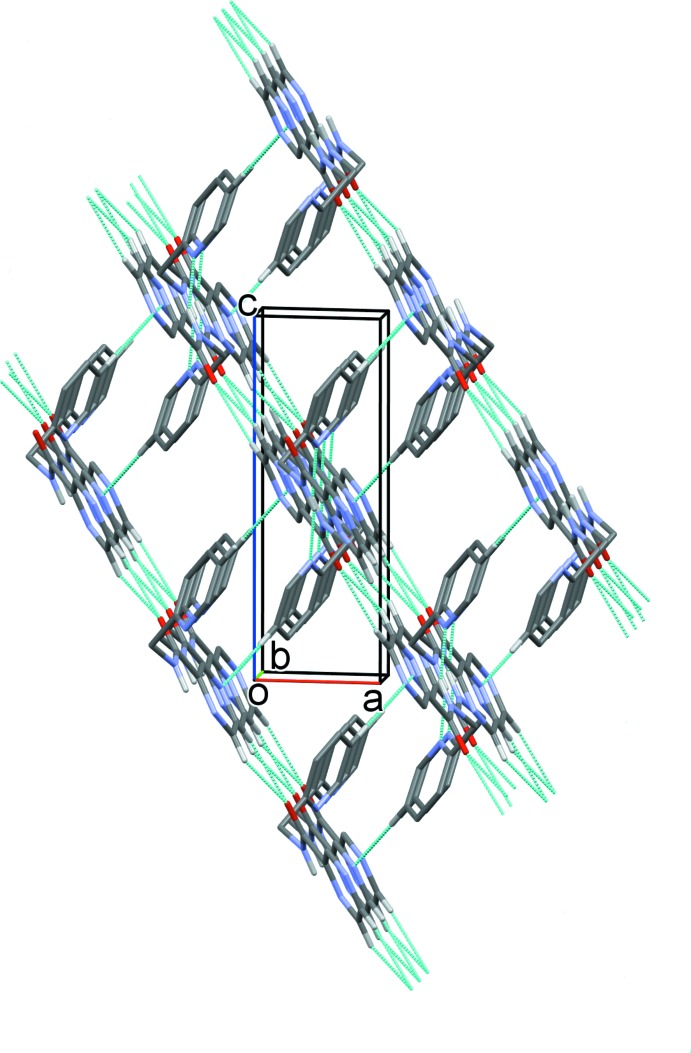
The crystal packing of compound H*L*1 viewed along the *b* axis. The N—H⋯N, C—H⋯O and C—H⋯N hydrogen bonds are shown as dashed lines (see Table 1 ▸ for details).

In the crystal of H*L*1, mol­ecules are linked by N—H⋯N hydrogen bonds, forming inversion dimers with an 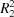
(10) ring motif. The dimers are linked *via* bifurcated-acceptor C—H⋯O hydrogen bonds, forming sheets lying parallel to (102) (see Table 1 ▸ and Fig. 3 ▸). The sheets are linked *via* C—H⋯N hydrogen bonds, forming a three-dimensional structure (Table 1 ▸ and Fig. 4 ▸).

**Figure 5:**
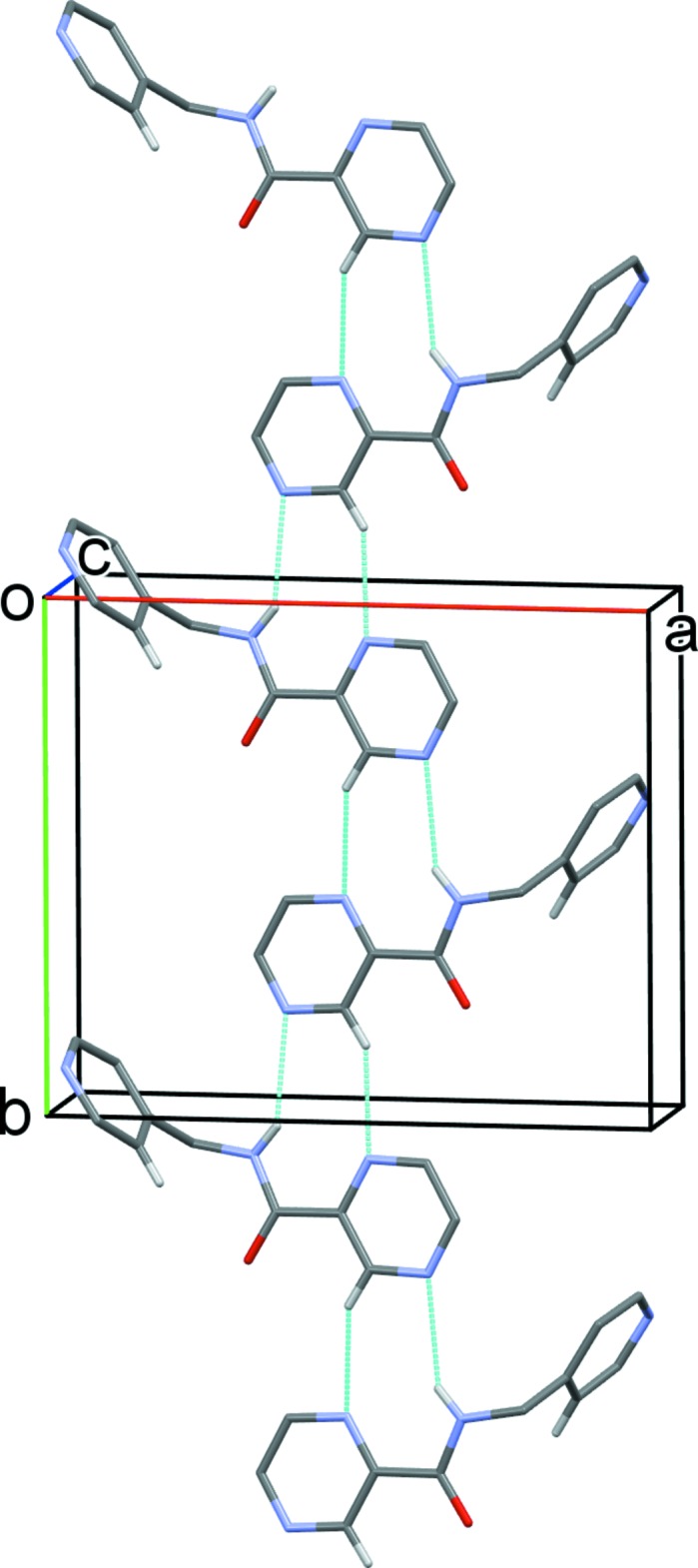
A partial view along the *c* axis of the crystal packing of compound H*L*2. The N—H⋯N, and C—H⋯N hydrogen bonds are shown as dashed lines (see Table 2 ▸ for details).

**Figure 6:**
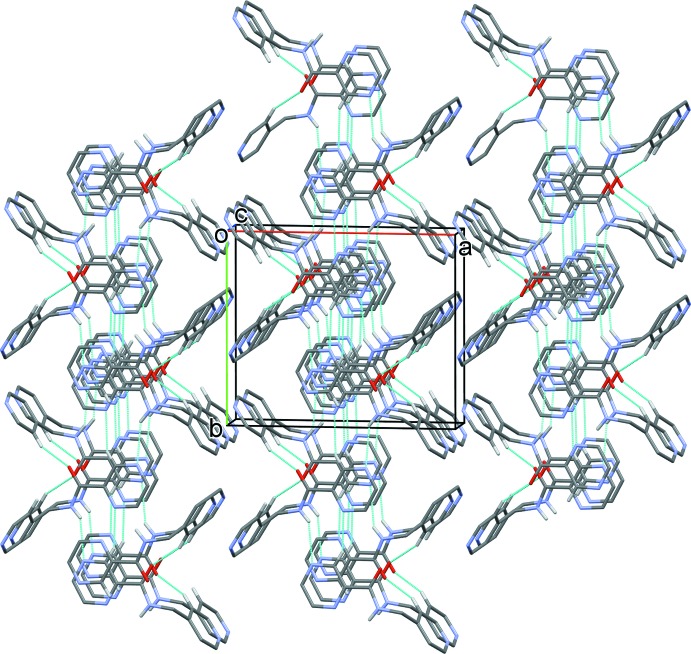
The crystal packing of compound H*L*2 viewed along the *c* axis. The N—H⋯N, C—H⋯O and C—H⋯N hydrogen bonds are shown as dashed lines (see Table 2 ▸ for details).

In the crystal of H*L*2, mol­ecules are linked by N—H⋯N and C—H⋯N hydrogen bonds to form chains propagating along [010], as shown in Table 2 ▸ and Fig. 5 ▸. The chains are linked *via* C—H⋯O hydrogen bonds, forming sheets lying parallel to (100). Within the sheets there are π–π inter­actions involving neighbouring pyrazine rings [*Cg*1⋯*Cg*1^i^ = 3.7113 (15) Å; *Cg*1 is the centroid of the pyrazine ring N1/N2/C1–C4; symmetry code: (i) = *x*, −*y* + 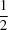, *z* − 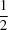]. The sheets are linked *via* slipped parallel π–π inter­actions involving inversion-related pyridine rings [*Cg*2⋯*Cg*2^ii^ = 3.6395 (11) Å, normal distance = 3.4164 (11), slippage = 1.255 Å; *Cg*2 is the centroid of pyridine ring N4/C7–C11; symmetry code: (ii) −*x*, −*y*, −*z*], forming a three-dimensional structure (Table 2 ▸ and Fig. 6 ▸).

## Database survey   

A search of the Cambridge Structural Database (Version 5.35, last update November 2013; Allen, 2002 ▸) indicated the presence of 282 structures containing the pyrazine-2-carboxamide unit. 81 of these concern pyrazine-2-carboxamide itself. There were 10 hits for complexes of ligand H*L*1. These include a cobalt(III) (Hellyer *et al.*, 2009 ▸), a chromium(III) (Khavasi *et al.*, 2010 ▸) and four copper(II) complexes (Mohamadou *et al.*, 2012 ▸; Khavasi *et al.*, 2011 ▸), all of which are mononuclear with the ligand coordinating in a tridentate manner. There are also two polymeric mercury chloride complexes (Khavasi & Sadegh, 2010 ▸), a binuclear manganese chloride complex (Khavasi *et al.*, 2009 ▸), and a polymeric silver tetra­fluoro­borate complex (Hellyer *et al.*, 2009 ▸), where the ligand coordinates in a bis-monodentate manner. Plus the report of the ligand itself as mentioned above (Sasan *et al.*, 2008 ▸). For ligand H*L*2 there were no hits.

## Synthesis and crystallization   

The precursor pyrazine-2-carb­oxy­lic acid methyl ester (2-pze) was prepared following the procedure described by Alvarez-Ibarra *et al.* (1994 ▸). 6.21 g (50 mmol) of pyrazine-2-carb­oxy­lic acid were added to 50 ml of absolute methanol in a two-necked flask (100 ml). The mixture was heated to 303 K and then 0.4 ml of concentrated sulfuric acid was added slowly. The mixture was heated for 23 h, at least. It was then poured over ice and made alkaline using NaOH (2 *N*), then extracted with CH_2_Cl_2_. The organic extract was dried over Na_2_SO_4_. The resulting yellow product was purified by recrystallization from hexane, or by column chromatography on silica gel using CH_2_Cl_2_ as eluant, giving finally colourless crystals (yield 80%).

The ligand H*L*1 was prepared by refluxing 2-pze (1.80 g, 13 mmol) and an excess of 2-(amino­meth­yl)pyridine (1.84 g, 17 mmol) in 12 ml of methanol, for 6 h in a two-necked flask (50 ml). A yellowish oil remained when the methanol was evaporated off. The excess of 2-(amino­meth­yl)pyridine was eliminated by column chromatography on silica gel using CH_2_Cl_2_ as eluant (*r* = 2 cm, *l* = 8 cm). A yellow band of 2-(amino­meth­yl)pyridine remained on the column. After evaporation, the ligand could be recrystallized from diethyl ether, aceto­nitrile or ethyl acetate. H*L*1 is very soluble in MeOH and in CH_2_Cl_2_. Recrystallization from diethyl ether gave colourless blocks of H*L*1 (yield 91%; m.p. 388 K). Analysis for C_11_H_10_N_4_O (*M_r_* = 214.46 g/mol) Calculated (%) C: 61.67 H: 4.71 N: 26.15; Found (%) C: 61.80 H: 4.76 N: 26.45. Spectroscopic data are available in the supporting information.

H*L*2 was prepared using the same procedure as for H*L*1. 2-pze (1.38 g, 10 mmol) with, this time, an excess of 4-(amino­meth­yl)pyridine (1.73 g, 16 mmol) were refluxed in 20 ml of methanol, for 20 h in a two-necked flask (50 ml). 4-(amino­meth­yl)pyridine (1g, 10 mmol) was then added to the red solution. After 4 h the solution was evaporated to about 8 ml. H*L*2 crystallized out at room temperature. About 20 ml of diethyl ether was added to filtrate the product. It was then recrystallized from a mixture of 3 ml of methanol and 40 ml of diethyl ether to give colourless blocks (yield 84%; m.p. 422 K). Anal. for C_11_H_10_N_4_O (*M_r_* = 214.46 g/mol) Calculated (%) C: 61.67 H: 4.71 N: 26.15 Found (%) C: 61.57 H: 4.75 N: 26.20. Spectroscopic data are available in the supporting information.

## Refinement   

Crystal data, data collection and structure refinement details are summarized in Table 3 ▸. The NH H atoms were located in difference Fourier maps and freely refined. The C-bound H atoms were included in calculated positions and treated as riding atoms: C—H = 0.95 Å for H*L*1 and = 0.93 Å for H*L*2, with *U*_iso_(H) = 1.2*U*_eq_(C).

## Supplementary Material

Crystal structure: contains datablock(s) HL1, HL2, global. DOI: 10.1107/S1600536814009519/hb0007sup1.cif

Structure factors: contains datablock(s) HL1. DOI: 10.1107/S1600536814009519/hb0007HL1sup2.hkl

Supporting information file. DOI: 10.1107/S1600536814009519/hb0007HL1sup4.cml

Structure factors: contains datablock(s) HL2. DOI: 10.1107/S1600536814009519/hb0007HL2sup3.hkl

Supporting information file. DOI: 10.1107/S1600536814009519/hb0007HL2sup5.cml

CCDC references: 1004272, 1004273

Additional supporting information:  crystallographic information; 3D view; checkCIF report

## Figures and Tables

**Table 1:** Hydrogen-bond geometry (Å, °) for H*L*1

*D*—H⋯*A*	*D*—H	H⋯*A*	*D*⋯*A*	*D*—H⋯*A*
N3—H3*N*⋯N1	0.901 (16)	2.332 (15)	2.7136 (15)	105.4 (11)
N3—H3*N*⋯N4^i^	0.901 (16)	2.206 (16)	2.9929 (14)	145.6 (13)
C3—H3⋯O1^ii^	0.95	2.51	3.1544 (15)	125
C4—H4⋯O1^ii^	0.95	2.56	3.1748 (15)	123
C10—H10⋯N2^iii^	0.95	2.62	3.5678 (17)	174

Symmetry codes: (i) 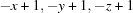; (ii) 
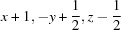
; (iii) 
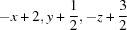
.

**Table 2:** Hydrogen-bond geometry (Å, °) for H*L*2

*D*—H⋯*A*	*D*—H	H⋯*A*	*D*⋯*A*	*D*—H⋯*A*
N3—H3*N*⋯N1	0.83 (3)	2.27 (3)	2.713 (3)	114 (2)
N3—H3*N*⋯N2^i^	0.83 (3)	2.52 (3)	3.214 (3)	142 (2)
C2—H2⋯N1^ii^	0.93	2.47	3.315 (3)	151
C8—H8⋯O1^iii^	0.93	2.55	3.373 (3)	148

Symmetry codes: (i) 
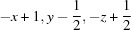
; (ii) 
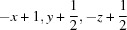
; (iii) 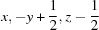.

**Table 3:** Experimental details

	H*L*1	H*L*2
Crystal data		
Chemical formula	C_11_H_10_N_4_O	C_11_H_10_N_4_O
*M*_r_	214.23	214.23
Crystal system, space group	Monoclinic, *P*2_1_/*c*	Monoclinic, *P*2_1_/*c*
Temperature (K)	153	293
*a*, *b*, *c* (Å)	4.1527 (4), 20.4629 (18), 12.0106 (11)	13.8564 (14), 11.1841 (11), 6.9122 (10)
β (°)	91.461 (11)	104.356 (14)
*V* (Å^3^)	1020.28 (16)	1037.7 (2)
*Z*	4	4
Radiation type	Mo *K*α	Mo *K*α
μ (mm^−1^)	0.10	0.09
Crystal size (mm)	0.50 × 0.40 × 0.35	0.38 × 0.30 × 0.19

Data collection		
Diffractometer	Stoe *IPDS* 1	Stoe AED2 four-circle
No. of measured, independent and observed [*I* > 2σ(*I*)] reflections	7822, 1958, 1548	4132, 1937, 1198
*R*_int_	0.035	0.032
(sin θ/λ)_max_ (Å^−1^)	0.615	0.605

Refinement		
*R*[*F* ^2^ > 2σ(*F* ^2^)], *wR*(*F* ^2^), *S*	0.032, 0.088, 1.03	0.054, 0.127, 1.10
No. of reflections	1958	1937
No. of parameters	149	150
H-atom treatment	H atoms treated by a mixture of independent and constrained refinement	H atoms treated by a mixture of independent and constrained refinement
Δρ_max_, Δρ_min_ (e Å^−3^)	0.17, −0.17	0.17, −0.16

Computer programs: *EXPOSE*, *CELL* and *INTEGRATE* in *IPDSI*, *STADI4* and *X-RED* (Stoe & Cie, 1997 ▸), *SHELXS97* and *SHELXL2013* (Sheldrick, 2008 ▸), *Mercury* (Macrae *et al.*, 2008 ▸), *PLATON* (Spek, 2009 ▸) and *publCIF* (Westrip, 2010 ▸).

## supplementary crystallographic information

### (HL1) N-(Pyridin-2-ylmethyl)pyrazine-2-carboxamide Crystal data

**Table:**

C_11_H_10_N_4_O	*F*(000) = 448
*M**_r_* = 214.23	*D*_x_ = 1.395 Mg m^−^^3^
Monoclinic, *P*2_1_/*c*	Mo *K*α radiation, λ = 0.71073 Å
*a* = 4.1527 (4) Å	Cell parameters from 6532 reflections
*b* = 20.4629 (18) Å	θ = 2.0–25.9°
*c* = 12.0106 (11) Å	µ = 0.10 mm^−^^1^
β = 91.461 (11)°	*T* = 153 K
*V* = 1020.28 (16) Å^3^	Block, colourless
*Z* = 4	0.50 × 0.40 × 0.35 mm

### (HL1) N-(Pyridin-2-ylmethyl)pyrazine-2-carboxamide Data collection

**Table:**

Stoe IPDS 1 diffractometer	1548 reflections with *I* > 2σ(*I*)
Radiation source: fine-focus sealed tube	*R*_int_ = 0.035
Plane graphite monochromator	θ_max_ = 25.9°, θ_min_ = 2.0°
φ rotation scans	*h* = −5→5
7822 measured reflections	*k* = −25→25
1958 independent reflections	*l* = −14→14

### (HL1) N-(Pyridin-2-ylmethyl)pyrazine-2-carboxamide Refinement

**Table:**

Refinement on *F*^2^	Primary atom site location: structure-invariant direct methods
Least-squares matrix: full	Secondary atom site location: difference Fourier map
*R*[*F*^2^ > 2σ(*F*^2^)] = 0.032	Hydrogen site location: mixed
*wR*(*F*^2^) = 0.088	H atoms treated by a mixture of independent and constrained refinement
*S* = 1.03	*w* = 1/[σ^2^(*F*_o_^2^) + (0.0539*P*)^2^ + 0.0704*P*] where *P* = (*F*_o_^2^ + 2*F*_c_^2^)/3
1958 reflections	(Δ/σ)_max_ < 0.001
149 parameters	Δρ_max_ = 0.17 e Å^−^^3^
0 restraints	Δρ_min_ = −0.17 e Å^−^^3^

### (HL1) N-(Pyridin-2-ylmethyl)pyrazine-2-carboxamide Special details

**Table:**

Experimental. Spectrosopic data for H*L*1: ^1^H NMR (400 MHz, DMSO-d6; code Hh/NH, Hl/C3, Hn/C1, Hm/C2, Hb/C11, Hd/C9, He/C8, Hc/C10, Hg/C6): 9.50 (t, 1H, Jhg = 6.0, Hh); 9.23 (d, 1H, Jlm = 1.5, Hl); 8.91 (d, 1H, Jnm = 2.5, Hn); 8.78 (dd, 1H, Jmn = 2.5, Jml = 1.5, Hm); 8.53 (ddd, 1H, Jbc = 4.8, Jbd = 1.8, Jbe = 0.8, Hb); 7.76 (td, 1H, Jdc = 7.7, Jdb = 1.8, Hd); 7.35 (d, 1H, Jed = 7.9, He); 7.28 (dd, 1H, Jcd = 7.7, Jcb = 4.8, Hc); 4.64 (d, 2H, Jgh = 6.0, He). ^13^C NMR (400 MHz, DMSO-d6): 163.9, 158.6, 149.7, 148.6, 145.5, 144.4, 144.3, 137.6, 123.1, 121.9, 45.0. IR (KBr pellet, cm^-1^): 3248 (*m*), 3066 (w), 3018 (w), 2949 (w), 1669 (*versus*), 1593 (*m*), 1570 (*m*), 1517 (*versus*), 1478 (*m*), 1462 (*s*), 1440 (*s*), 1423 (*s*), 1389 (*s*), 1350 (*m*), 1320 (*m*), 1287 (*s*), 1250 (*m*), 1221 (*m*), 1168 (*s*), 1148 (*m*), 1103 (*m*), 1055 (*m*), 1021 (*s*), 1000 (*m*), 975 (*m*), 870 (*m*), 840 (w), 776 (*m*), 753 (*s*), 707 (*m*), 634 (*m*), 611 (*m*), 528 (*m*), 444 (*m*).
Geometry. All e.s.d.'s (except the e.s.d. in the dihedral angle between two l.s. planes) are estimated using the full covariance matrix. The cell e.s.d.'s are taken into account individually in the estimation of e.s.d.'s in distances, angles and torsion angles; correlations between e.s.d.'s in cell parameters are only used when they are defined by crystal symmetry. An approximate (isotropic) treatment of cell e.s.d.'s is used for estimating e.s.d.'s involving l.s. planes.

### (HL1) N-(Pyridin-2-ylmethyl)pyrazine-2-carboxamide Fractional atomic coordinates and isotropic or equivalent isotropic displacement parameters (Å^2^)

**Table:**

	*x*	*y*	*z*	*U*_iso_*/*U*_eq_	
N1	0.7345 (3)	0.30055 (5)	0.42625 (8)	0.0260 (2)	
N2	0.7780 (3)	0.16917 (5)	0.49125 (9)	0.0343 (3)	
N3	0.4290 (2)	0.38859 (5)	0.55546 (8)	0.0224 (2)	
H3N	0.514 (4)	0.3982 (7)	0.4892 (13)	0.037 (4)*	
N4	0.5222 (3)	0.53951 (5)	0.66045 (8)	0.0293 (3)	
O1	0.3074 (2)	0.30857 (4)	0.67870 (7)	0.0344 (2)	
C1	0.6100 (3)	0.27903 (5)	0.52119 (9)	0.0215 (3)	
C2	0.6318 (3)	0.21416 (6)	0.55317 (10)	0.0287 (3)	
H2	0.5401	0.2011	0.6213	0.034*	
C3	0.9010 (3)	0.19099 (6)	0.39702 (10)	0.0305 (3)	
H3	1.0071	0.1609	0.3501	0.037*	
C4	0.8800 (3)	0.25567 (6)	0.36486 (10)	0.0290 (3)	
H4	0.9724	0.2686	0.2967	0.035*	
C5	0.4355 (3)	0.32697 (6)	0.59277 (9)	0.0229 (3)	
C6	0.2435 (3)	0.43932 (6)	0.60984 (10)	0.0263 (3)	
H6A	0.0627	0.4186	0.6488	0.032*	
H6B	0.1503	0.4688	0.5522	0.032*	
C7	0.4391 (3)	0.47934 (5)	0.69249 (9)	0.0219 (3)	
C8	0.5266 (3)	0.45428 (6)	0.79615 (10)	0.0271 (3)	
H8	0.4640	0.4113	0.8163	0.033*	
C9	0.7051 (3)	0.49220 (6)	0.86977 (10)	0.0312 (3)	
H9	0.7682	0.4757	0.9410	0.037*	
C10	0.7906 (4)	0.55455 (6)	0.83801 (11)	0.0350 (3)	
H10	0.9115	0.5822	0.8870	0.042*	
C11	0.6957 (4)	0.57556 (6)	0.73353 (11)	0.0376 (3)	
H11	0.7569	0.6183	0.7117	0.045*	

### (HL1) N-(Pyridin-2-ylmethyl)pyrazine-2-carboxamide Atomic displacement parameters (Å^2^)

**Table:**

	*U*^11^	*U*^22^	*U*^33^	*U*^12^	*U*^13^	*U*^23^
N1	0.0305 (6)	0.0246 (5)	0.0231 (5)	−0.0011 (4)	0.0045 (4)	−0.0005 (4)
N2	0.0434 (7)	0.0239 (5)	0.0356 (6)	0.0046 (5)	0.0043 (5)	−0.0006 (4)
N3	0.0263 (5)	0.0207 (5)	0.0201 (5)	0.0007 (4)	0.0020 (4)	−0.0008 (4)
N4	0.0412 (7)	0.0219 (5)	0.0249 (5)	0.0016 (4)	0.0022 (4)	0.0007 (4)
O1	0.0454 (6)	0.0295 (5)	0.0288 (5)	0.0000 (4)	0.0154 (4)	0.0036 (4)
C1	0.0219 (6)	0.0217 (6)	0.0208 (5)	−0.0024 (5)	−0.0014 (4)	−0.0008 (4)
C2	0.0369 (8)	0.0247 (6)	0.0246 (6)	0.0006 (5)	0.0032 (5)	0.0016 (5)
C3	0.0327 (7)	0.0275 (6)	0.0313 (7)	0.0029 (5)	0.0039 (5)	−0.0067 (5)
C4	0.0321 (7)	0.0305 (6)	0.0246 (6)	−0.0012 (5)	0.0056 (5)	−0.0028 (5)
C5	0.0233 (6)	0.0242 (6)	0.0211 (6)	−0.0021 (5)	−0.0003 (5)	−0.0001 (4)
C6	0.0244 (7)	0.0248 (6)	0.0296 (6)	0.0062 (5)	−0.0010 (5)	−0.0021 (5)
C7	0.0210 (6)	0.0213 (6)	0.0235 (6)	0.0055 (5)	0.0057 (4)	−0.0011 (4)
C8	0.0275 (7)	0.0258 (6)	0.0281 (6)	0.0005 (5)	0.0024 (5)	0.0046 (5)
C9	0.0333 (7)	0.0374 (7)	0.0228 (6)	0.0044 (6)	−0.0001 (5)	0.0010 (5)
C10	0.0420 (8)	0.0310 (7)	0.0317 (7)	−0.0002 (6)	−0.0034 (6)	−0.0093 (5)
C11	0.0554 (9)	0.0207 (6)	0.0366 (7)	−0.0046 (6)	−0.0022 (6)	−0.0006 (5)

### (HL1) N-(Pyridin-2-ylmethyl)pyrazine-2-carboxamide Geometric parameters (Å, º)

**Table:**

N1—C4	1.3323 (16)	C3—H3	0.9500
N1—C1	1.3386 (15)	C4—H4	0.9500
N2—C3	1.3307 (17)	C6—C7	1.5082 (16)
N2—C2	1.3387 (16)	C6—H6A	0.9900
N3—C5	1.3382 (15)	C6—H6B	0.9900
N3—C6	1.4570 (15)	C7—C8	1.3865 (17)
N3—H3N	0.901 (16)	C8—C9	1.3784 (18)
N4—C7	1.3379 (15)	C8—H8	0.9500
N4—C11	1.3422 (17)	C9—C10	1.3806 (18)
O1—C5	1.2319 (14)	C9—H9	0.9500
C1—C2	1.3842 (16)	C10—C11	1.3745 (19)
C1—C5	1.5028 (16)	C10—H10	0.9500
C2—H2	0.9500	C11—H11	0.9500
C3—C4	1.3808 (18)		
			
C4—N1—C1	115.80 (10)	N3—C6—C7	113.57 (9)
C3—N2—C2	115.53 (11)	N3—C6—H6A	108.9
C5—N3—C6	121.84 (10)	C7—C6—H6A	108.9
C5—N3—H3N	119.7 (9)	N3—C6—H6B	108.9
C6—N3—H3N	117.7 (9)	C7—C6—H6B	108.9
C7—N4—C11	117.12 (10)	H6A—C6—H6B	107.7
N1—C1—C2	121.86 (11)	N4—C7—C8	122.34 (11)
N1—C1—C5	118.36 (10)	N4—C7—C6	116.69 (10)
C2—C1—C5	119.76 (10)	C8—C7—C6	120.97 (11)
N2—C2—C1	122.19 (11)	C9—C8—C7	119.49 (11)
N2—C2—H2	118.9	C9—C8—H8	120.3
C1—C2—H2	118.9	C7—C8—H8	120.3
N2—C3—C4	122.47 (11)	C8—C9—C10	118.73 (11)
N2—C3—H3	118.8	C8—C9—H9	120.6
C4—C3—H3	118.8	C10—C9—H9	120.6
N1—C4—C3	122.14 (11)	C11—C10—C9	118.14 (12)
N1—C4—H4	118.9	C11—C10—H10	120.9
C3—C4—H4	118.9	C9—C10—H10	120.9
O1—C5—N3	124.27 (11)	N4—C11—C10	124.17 (12)
O1—C5—C1	120.30 (10)	N4—C11—H11	117.9
N3—C5—C1	115.43 (10)	C10—C11—H11	117.9
			
C4—N1—C1—C2	0.03 (17)	C2—C1—C5—N3	179.54 (11)
C4—N1—C1—C5	−178.51 (11)	C5—N3—C6—C7	95.48 (13)
C3—N2—C2—C1	0.06 (19)	C11—N4—C7—C8	−0.18 (18)
N1—C1—C2—N2	−0.1 (2)	C11—N4—C7—C6	179.86 (11)
C5—C1—C2—N2	178.43 (11)	N3—C6—C7—N4	104.41 (12)
C2—N2—C3—C4	0.03 (19)	N3—C6—C7—C8	−75.55 (14)
C1—N1—C4—C3	0.05 (18)	N4—C7—C8—C9	0.12 (18)
N2—C3—C4—N1	−0.1 (2)	C6—C7—C8—C9	−179.92 (11)
C6—N3—C5—O1	−5.16 (19)	C7—C8—C9—C10	0.33 (19)
C6—N3—C5—C1	174.05 (10)	C8—C9—C10—C11	−0.7 (2)
N1—C1—C5—O1	177.36 (11)	C7—N4—C11—C10	−0.2 (2)
C2—C1—C5—O1	−1.21 (18)	C9—C10—C11—N4	0.6 (2)
N1—C1—C5—N3	−1.88 (16)		

### (HL1) N-(Pyridin-2-ylmethyl)pyrazine-2-carboxamide Hydrogen-bond geometry (Å, º)

**Table:**

*D*—H···*A*	*D*—H	H···*A*	*D*···*A*	*D*—H···*A*
N3—H3*N*···N1	0.901 (16)	2.332 (15)	2.7136 (15)	105.4 (11)
N3—H3*N*···N4^i^	0.901 (16)	2.206 (16)	2.9929 (14)	145.6 (13)
C3—H3···O1^ii^	0.95	2.51	3.1544 (15)	125
C4—H4···O1^ii^	0.95	2.56	3.1748 (15)	123
C10—H10···N2^iii^	0.95	2.62	3.5678 (17)	174

Symmetry codes: (i) −*x*+1, −*y*+1, −*z*+1; (ii) *x*+1, −*y*+1/2, *z*−1/2; (iii) −*x*+2, *y*+1/2, −*z*+3/2.

### (HL2) N-(Pyridin-4-ylmethyl)pyrazine-2-carboxamide Crystal data

**Table:**

C_11_H_10_N_4_O	*F*(000) = 448
*M**_r_* = 214.23	*D*_x_ = 1.371 Mg m^−^^3^
Monoclinic, *P*2_1_/*c*	Mo *K*α radiation, λ = 0.71073 Å
*a* = 13.8564 (14) Å	Cell parameters from 20 reflections
*b* = 11.1841 (11) Å	θ = 10.4–17.6°
*c* = 6.9122 (10) Å	µ = 0.09 mm^−^^1^
β = 104.356 (14)°	*T* = 293 K
*V* = 1037.7 (2) Å^3^	Block, colourless
*Z* = 4	0.38 × 0.30 × 0.19 mm

### (HL2) N-(Pyridin-4-ylmethyl)pyrazine-2-carboxamide Data collection

**Table:**

Stoe AED2 four-circle diffractometer	*R*_int_ = 0.032
Radiation source: fine-focus sealed tube	θ_max_ = 25.5°, θ_min_ = 2.4°
Plane graphite monochromator	*h* = −16→16
2θ/ω scans	*k* = −13→0
4132 measured reflections	*l* = −8→8
1937 independent reflections	2 standard reflections every 60 min
1198 reflections with *I* > 2σ(*I*)	intensity decay: 2%

### (HL2) N-(Pyridin-4-ylmethyl)pyrazine-2-carboxamide Refinement

**Table:**

Refinement on *F*^2^	Hydrogen site location: mixed
Least-squares matrix: full	H atoms treated by a mixture of independent and constrained refinement
*R*[*F*^2^ > 2σ(*F*^2^)] = 0.054	*w* = 1/[σ^2^(*F*_o_^2^) + (0.0426*P*)^2^ + 0.1904*P*] where *P* = (*F*_o_^2^ + 2*F*_c_^2^)/3
*wR*(*F*^2^) = 0.127	(Δ/σ)_max_ < 0.001
*S* = 1.10	Δρ_max_ = 0.17 e Å^−^^3^
1937 reflections	Δρ_min_ = −0.16 e Å^−^^3^
150 parameters	Extinction correction: SHELXL2013 (Sheldrick, 2008), Fc^*^=kFc[1+0.001xFc^2^λ^3^/sin(2θ)]^-1/4^
0 restraints	Extinction coefficient: 0.014 (2)

### (HL2) N-(Pyridin-4-ylmethyl)pyrazine-2-carboxamide Special details

**Table:**

Experimental. Spectroscopic data for H*L*2: ^1^H NMR (400 MHz, DMSO-d6; code Hh/NH, Hl/C3, Hn/C1, Hm/C2, Hb/C11, Hd/C9, Ha/C11/, He/C8, Hg/C6): 9.63 (t, 1H, Jhg = 6.3, Hh); 9.21 (d, 1H, Jlm = 1.5, Hl); 8.90 (d, 1H, Jnm = 2.5, Hn); 8.77 (dd, 1H, Jmn = 2.5, Jml = 1.5, Hm); 8.49 (dd, 2H, Jba = 4.4, Jbe = 1.6, Hb = Hd); 7.31 (dd, 2H, Jab = 4.4, Jad = 1.6, Ha = He); 4.54 (d, 2H, Jgh = 6.3, Hg). ^13^C NMR (400 MHz, DMSO-d6): 164.2, 150.4, 149.0, 148.5, 145.4, 144.5, 144.3, 123.0, 42.4. IR (KBr pellet, cm^-1^): 3366 (*versus*), 3089 (*s*), 3050 (*m*), 3031 (*s*), 2967 (*m*), 2935 (*s*), 1966 (w), 1924 (w), 1834 (w), 1674 (*versus*), 1634 (*s*), 1602 (*versus*), 1585 (*s*), 1564 (*s*) 1526 (*versus*), 1467 (*versus*), 1429 (*versus*), 1415 (*versus*), 1399 (*versus*), 1359 (*s*), 1329 (*s*), 1288 (*versus*), 1240 (*m*), 1216 (*versus*), 1167 (*s*), 1155 (*s*), 1059 (*versus*), 1025 (*versus*), 1020 (*versus*), 993 (*s*), 981 (*s*), 968 (*s*), 887 (*m*), 870 (*s*), 842 (*s*), 832 (*s*), 804 (*versus*), 775 (*s*), 731 (*m*), 652 (*versus*), 608 (*s*), 517 (*s*), 479 (*m*), 445 (*versus*), 411 (*m*).
Geometry. All e.s.d.'s (except the e.s.d. in the dihedral angle between two l.s. planes) are estimated using the full covariance matrix. The cell e.s.d.'s are taken into account individually in the estimation of e.s.d.'s in distances, angles and torsion angles; correlations between e.s.d.'s in cell parameters are only used when they are defined by crystal symmetry. An approximate (isotropic) treatment of cell e.s.d.'s is used for estimating e.s.d.'s involving l.s. planes.

### (HL2) N-(Pyridin-4-ylmethyl)pyrazine-2-carboxamide Fractional atomic coordinates and isotropic or equivalent isotropic displacement parameters (Å^2^)

**Table:**

	*x*	*y*	*z*	*U*_iso_*/*U*_eq_	
O1	0.32038 (14)	0.28041 (15)	0.2248 (3)	0.0618 (6)	
N1	0.52051 (15)	0.08089 (17)	0.2297 (3)	0.0456 (6)	
N2	0.61838 (17)	0.29856 (19)	0.2153 (3)	0.0530 (6)	
N3	0.32823 (16)	0.0787 (2)	0.2548 (3)	0.0495 (6)	
H3N	0.367 (2)	0.022 (2)	0.251 (4)	0.057 (9)*	
N4	0.04496 (18)	−0.1046 (2)	−0.2659 (4)	0.0677 (7)	
C1	0.47371 (18)	0.1855 (2)	0.2255 (3)	0.0389 (6)	
C2	0.5226 (2)	0.2927 (2)	0.2174 (4)	0.0477 (7)	
H2	0.4870	0.3635	0.2132	0.057*	
C3	0.6641 (2)	0.1935 (2)	0.2189 (4)	0.0531 (7)	
H3	0.7309	0.1925	0.2166	0.064*	
C4	0.61612 (19)	0.0861 (2)	0.2258 (4)	0.0510 (7)	
H4	0.6515	0.0153	0.2279	0.061*	
C5	0.36661 (19)	0.1863 (2)	0.2342 (4)	0.0442 (6)	
C6	0.22661 (19)	0.0590 (2)	0.2660 (4)	0.0548 (7)	
H6A	0.1972	0.1350	0.2877	0.066*	
H6B	0.2267	0.0080	0.3795	0.066*	
C7	0.16399 (17)	0.0020 (2)	0.0798 (4)	0.0431 (6)	
C8	0.1571 (2)	0.0498 (2)	−0.1071 (4)	0.0534 (7)	
H8	0.1922	0.1187	−0.1216	0.064*	
C9	0.0979 (2)	−0.0052 (3)	−0.2711 (5)	0.0652 (8)	
H9	0.0943	0.0291	−0.3953	0.078*	
C10	0.0528 (2)	−0.1497 (2)	−0.0853 (5)	0.0627 (8)	
H10	0.0171	−0.2188	−0.0752	0.075*	
C11	0.11055 (19)	−0.1010 (2)	0.0887 (4)	0.0517 (7)	
H11	0.1134	−0.1374	0.2110	0.062*	

### (HL2) N-(Pyridin-4-ylmethyl)pyrazine-2-carboxamide Atomic displacement parameters (Å^2^)

**Table:**

	*U*^11^	*U*^22^	*U*^33^	*U*^12^	*U*^13^	*U*^23^
O1	0.0615 (13)	0.0417 (11)	0.0868 (15)	0.0116 (10)	0.0272 (11)	−0.0013 (10)
N1	0.0459 (13)	0.0334 (12)	0.0586 (14)	0.0012 (10)	0.0149 (10)	−0.0024 (10)
N2	0.0553 (15)	0.0427 (14)	0.0610 (15)	−0.0100 (11)	0.0144 (11)	−0.0006 (11)
N3	0.0421 (14)	0.0421 (14)	0.0663 (16)	−0.0008 (11)	0.0170 (11)	−0.0043 (11)
N4	0.0590 (16)	0.0684 (18)	0.0749 (19)	0.0011 (13)	0.0148 (14)	−0.0111 (15)
C1	0.0441 (14)	0.0323 (13)	0.0399 (14)	−0.0006 (11)	0.0101 (11)	−0.0028 (11)
C2	0.0560 (18)	0.0325 (15)	0.0531 (17)	0.0008 (12)	0.0109 (13)	−0.0023 (12)
C3	0.0488 (17)	0.0505 (16)	0.0607 (17)	−0.0050 (14)	0.0149 (13)	0.0007 (14)
C4	0.0496 (16)	0.0406 (15)	0.0646 (18)	0.0044 (13)	0.0174 (13)	−0.0019 (13)
C5	0.0501 (16)	0.0376 (14)	0.0447 (15)	0.0009 (13)	0.0117 (12)	−0.0045 (12)
C6	0.0471 (16)	0.0585 (18)	0.0625 (19)	−0.0012 (13)	0.0206 (14)	−0.0040 (14)
C7	0.0321 (13)	0.0416 (14)	0.0582 (18)	0.0048 (11)	0.0162 (12)	0.0016 (12)
C8	0.0442 (15)	0.0551 (17)	0.0646 (19)	−0.0022 (13)	0.0204 (14)	0.0059 (15)
C9	0.0589 (19)	0.084 (2)	0.056 (2)	0.0091 (17)	0.0214 (16)	0.0066 (17)
C10	0.0530 (18)	0.0444 (16)	0.092 (3)	−0.0008 (14)	0.0204 (17)	−0.0063 (17)
C11	0.0457 (15)	0.0439 (15)	0.0674 (19)	0.0043 (13)	0.0179 (13)	0.0087 (14)

### (HL2) N-(Pyridin-4-ylmethyl)pyrazine-2-carboxamide Geometric parameters (Å, º)

**Table:**

O1—C5	1.225 (3)	C3—H3	0.9300
N1—C4	1.333 (3)	C4—H4	0.9300
N1—C1	1.334 (3)	C6—C7	1.504 (3)
N2—C3	1.332 (3)	C6—H6A	0.9700
N2—C2	1.333 (3)	C6—H6B	0.9700
N3—C5	1.338 (3)	C7—C11	1.379 (3)
N3—C6	1.446 (3)	C7—C8	1.379 (4)
N3—H3N	0.83 (3)	C8—C9	1.369 (4)
N4—C10	1.325 (4)	C8—H8	0.9300
N4—C9	1.337 (4)	C9—H9	0.9300
C1—C2	1.385 (3)	C10—C11	1.381 (4)
C1—C5	1.500 (3)	C10—H10	0.9300
C2—H2	0.9300	C11—H11	0.9300
C3—C4	1.379 (3)		
			
C4—N1—C1	116.2 (2)	N3—C6—C7	112.4 (2)
C3—N2—C2	115.2 (2)	N3—C6—H6A	109.1
C5—N3—C6	124.1 (2)	C7—C6—H6A	109.1
C5—N3—H3N	113.8 (19)	N3—C6—H6B	109.1
C6—N3—H3N	121.9 (19)	C7—C6—H6B	109.1
C10—N4—C9	115.1 (3)	H6A—C6—H6B	107.9
N1—C1—C2	121.3 (2)	C11—C7—C8	116.9 (3)
N1—C1—C5	119.0 (2)	C11—C7—C6	121.2 (2)
C2—C1—C5	119.6 (2)	C8—C7—C6	121.9 (2)
N2—C2—C1	122.8 (2)	C9—C8—C7	119.3 (3)
N2—C2—H2	118.6	C9—C8—H8	120.3
C1—C2—H2	118.6	C7—C8—H8	120.3
N2—C3—C4	122.5 (3)	N4—C9—C8	124.8 (3)
N2—C3—H3	118.7	N4—C9—H9	117.6
C4—C3—H3	118.7	C8—C9—H9	117.6
N1—C4—C3	121.9 (2)	N4—C10—C11	124.4 (3)
N1—C4—H4	119.0	N4—C10—H10	117.8
C3—C4—H4	119.0	C11—C10—H10	117.8
O1—C5—N3	124.2 (2)	C7—C11—C10	119.5 (3)
O1—C5—C1	120.9 (2)	C7—C11—H11	120.3
N3—C5—C1	114.9 (2)	C10—C11—H11	120.3
			
C4—N1—C1—C2	0.1 (3)	C2—C1—C5—N3	−175.8 (2)
C4—N1—C1—C5	−178.6 (2)	C5—N3—C6—C7	108.0 (3)
C3—N2—C2—C1	0.9 (4)	N3—C6—C7—C11	126.7 (3)
N1—C1—C2—N2	−0.7 (4)	N3—C6—C7—C8	−53.3 (3)
C5—C1—C2—N2	178.0 (2)	C11—C7—C8—C9	0.8 (4)
C2—N2—C3—C4	−0.5 (4)	C6—C7—C8—C9	−179.2 (2)
C1—N1—C4—C3	0.3 (4)	C10—N4—C9—C8	0.0 (4)
N2—C3—C4—N1	−0.1 (4)	C7—C8—C9—N4	−0.3 (4)
C6—N3—C5—O1	0.9 (4)	C9—N4—C10—C11	−0.1 (4)
C6—N3—C5—C1	−179.9 (2)	C8—C7—C11—C10	−0.9 (4)
N1—C1—C5—O1	−177.8 (2)	C6—C7—C11—C10	179.1 (2)
C2—C1—C5—O1	3.4 (4)	N4—C10—C11—C7	0.6 (4)
N1—C1—C5—N3	2.9 (3)		

### (HL2) N-(Pyridin-4-ylmethyl)pyrazine-2-carboxamide Hydrogen-bond geometry (Å, º)

**Table:**

*D*—H···*A*	*D*—H	H···*A*	*D*···*A*	*D*—H···*A*
N3—H3*N*···N1	0.83 (3)	2.27 (3)	2.713 (3)	114 (2)
N3—H3*N*···N2^i^	0.83 (3)	2.52 (3)	3.214 (3)	142 (2)
C2—H2···N1^ii^	0.93	2.47	3.315 (3)	151
C8—H8···O1^iii^	0.93	2.55	3.373 (3)	148

Symmetry codes: (i) −*x*+1, *y*−1/2, −*z*+1/2; (ii) −*x*+1, *y*+1/2, −*z*+1/2; (iii) *x*, −*y*+1/2, *z*−1/2.
